# Health Service Use Among Young Adults With a History of Adolescent Cannabis Use

**DOI:** 10.1001/jamanetworkopen.2025.39977

**Published:** 2025-10-28

**Authors:** Pablo Martínez, Nicholas Chadi, Natalie Castellanos-Ryan, Francis Vergunst, Marc Dorais, Jean R. Séguin, Frank Vitaro, Caroline Temcheff, Richard E. Tremblay, Michel Boivin, Sylvana M. Côté, Marie-Claude Geoffroy, Massimiliano Orri

**Affiliations:** 1 McGill Group for Suicide Studies, Douglas Mental Health University Institute, Department of Psychiatry, McGill University, Montréal, Québec, Canada; 2 Azreli Research Centre, CHU Sainte-Justine, Montréal, Québec, Canada; 3 Department of Special Needs Education, University of Oslo, Oslo, Norway; 4 StatSciences Inc, Notre-Dame-de-l’Île-Perrot, Québec, Canada; 5 Department of Psychiatry and Addictology, Université de Montréal, Montréal, Québec, Canada; 6 School of Psychoeducation, Université de Montréal, Montréal, Québec, Canada; 7 Department of Educational and Counselling Psychology, McGill University, Montréal, Québec, Canada; 8 Department of Psychology, Université de Montréal, Montréal, Québec, Canada; 9 Department of Pediatrics, Université de Montréal, Montréal, Québec, Canada; 10 Department of Psychology, Université Laval, Québec City, Québec, Canada; 11 École de Santé Publique, Université de Montréal, Montréal, Québec, Canada; 12 Department of Epidemiology, Biostatistics, and Occupational Health, School of Population and Global Health, McGill University, Montréal, Québec, Canada; 13 Danish Research Institute for Suicide Prevention, Mental Health Centre Copenhagen, Copenhagen, Denmark

## Abstract

**Importance:**

Adolescent cannabis use is a substantial public health concern given its associations with adverse mental and physical health outcomes. Understanding how distinct use patterns are associated with medical care utilization in young adulthood is critical for prevention.

**Objective:**

To examine the association between patterns of adolescent cannabis use and medical care utilization for mental and physical health conditions in young adulthood.

**Design, Setting, and Participants:**

This population-based birth cohort study linked to population-wide administrative medical care databases was conducted in the Province of Québec, Canada. Participants included individuals recruited for the Québec Longitudinal Study of Child Development, followed up from birth (1997-1998) to 23 years of age (2021). Data were analyzed November 2023 to February 2025.

**Exposure:**

Self-reported past 12-month cannabis use at ages 12, 13, 15, and 17 years.

**Main Outcomes and Measures:**

Medical care utilization for any mental disorder (including common mental disorders and substance-related disorders), suicide-related behaviors, and any physical health condition (including respiratory diseases, injuries and poisoning, and other physical diseases) between ages 18 and 23 years. Analyses were adjusted for 32 individual, family, and community-level confounders measured from birth to younger than 12 years of age using overlap weights.

**Results:**

Data for 1591 individuals (818 female [51.4%]; mean [SD] age at first exposure assessment, 12.1 [0.3] years) were analyzed. Three distinct adolescent cannabis use patterns were identified via group-based trajectory modeling: nonuse (948 [59.6%]), late-onset use (318 [20.0%], initiating after age 15 years), and early-onset and frequent use (325 [20.4%], initiating before age 15 years). In fully adjusted analyses, individuals with early-onset and frequent use had significantly higher odds of medical care utilization for any mental disorder (odds ratio [OR], 1.51 [95% CI, 1.10-2.08]), common mental disorders (OR, 1.57 [95% CI, 1.12-2.21]), any physical health condition (OR, 1.86 [95% CI, 1.30-2.67]), injuries and poisoning (OR, 1.41 [1.05-1.89]), and other physical diseases (OR, 1.47 [95% CI, 1.08-1.98]), compared with individuals with no use. Individuals with late-onset use did not differ significantly from those with nonuse for mental health outcomes (OR, 1.13 [95% CI, 0.80-1.58]) but had higher odds of medical care utilization for any physical health condition (OR, 1.63 [95% CI, 1.16-2.28]).

**Conclusions and Relevance:**

Findings of this birth cohort study indicated that early-onset and frequent cannabis use was associated with greater medical care utilization for both mental and physical health conditions in young adulthood. These findings support the relevance of delaying initiation and reducing intensity of cannabis use during adolescence.

## Key Points

**Question:**

Is adolescent cannabis use associated with medical care utilization for mental and physical health conditions in young adulthood?

**Findings:**

In this cohort study of 1591 individuals followed up to age 23 years, adolescents who initiated cannabis use before 15 years of age and used frequently had increased odds of medical care utilization for both mental and physical conditions in young adulthood, compared with adolescents without cannabis use. Adolescents with late-onset use had no increased odds for mental health care utilization but did exhibit higher odds for physical conditions.

**Meaning:**

Evidence from this study indicated that early and frequent cannabis use was associated with medical care utilization later in life, underscoring the importance of delaying or reducing adolescent cannabis use.

## Teaser

This cohort study investigates the associations between distinct patterns of cannabis use in adolescence and medical care utilization for mental and physical health conditions in young adulthood.

## Introduction

Adolescence is a critical developmental period marked by risk-taking behaviors and heightened sensitivity to substance-related neurobiological changes.^1^ Cannabis is among the most commonly used substances by adolescents in Canada and worldwide.^2^ Meta-analytical evidence associates regular adolescent use of cannabis with increased odds of substance-related problems, suicidality, and depression in young adulthood.^3,4^ Early-onset use (before 15 years of age) has been associated with higher risk of psychosis,^5^ and longer-term use has been associated with physical health issues (eg, respiratory symptoms) that may emerge in adolescence.^6^ In Canada, where nonmedical cannabis was legalized in 2018, 12% of adolescents in 7th to 9th grade (typically 12 to 15 years of age) reported past-year cannabis use, highlighting the emergence of use during early adolescence.^7^ Widespread adolescent cannabis use, rising product potency,^8^ and increasing cannabis-related harms^9^ make adolescent cannabis use a substantial public health concern.^10^

Although not all adolescents are equally susceptible, a subset who begin using early and frequently appear especially vulnerable to subsequent health and social issues,^11^ reporting increased odds of anxiety and depression,^12,13,14,15,16,17^ higher risk of cannabis and alcohol-related harms,^12,13,14,15,16,18,19,20,21,22,23,24^ and worse physical health.^16,25^ Yet previous studies often overlook critical family and environmental factors measured in early childhood that may confound the association between adolescent cannabis use and health outcomes^22,24^ and primarily rely on self-reported health outcomes, which may not fully capture severe health conditions requiring medical care.

To address these gaps, we used data from the Québec Longitudinal Study of Child Development (QLSCD),^26^ a population-based birth cohort linked to population-wide administrative medical care databases in Québec, Canada. The QLSCD provides comprehensive early-life information at the individual, familial, and environmental level,^26^ while administrative data offer objective, detailed records of medical care utilization within a universal health care system. By clarifying how different adolescent cannabis use patterns are associated with subsequent medical care utilization for mental and physical health conditions in early adulthood, these findings may inform targeted prevention strategies and guide resource allocation to mitigate long-term harms.

## Methods

The QLSCD was approved by the Research Ethics Board of the Institut de la statistique du Québec (ISQ), Sainte Justine Hospital Research Centre and Montreal West Island Integrated University Health and Social Services Centre. Ethical approval for the overall linkage project was obtained by the Montreal West Island Integrated University Health and Social Services Centre’s Research Ethics Board. Analyses for this report were approved by McGill University’s Faculty of Medicine and Health Science Research Ethics Board. Written informed consent was obtained from participants or parents at each data collection. This study followed the Strengthening the Reporting of Observational Studies in Epidemiology (STROBE) reporting guideline for cohort studies.^27^

### Participants

Participants were drawn from the QLSCD, a population-based birth cohort of 2120 children born in Québec (1997-1998).^26^ The sampling strategy and eligibility criteria are described elsewhere.^26^ Data were collected at multiple time points from infancy to young adulthood, including waves at ages 0.5, 1.5, 2.5, 3.5, 4, 5, 6, 7, 8, 10, 12, 13, 15, and 17 years. The current study included data for 1591 participants (75.0% of the original cohort) who provided at least 1 data point on adolescent cannabis use between ages 12 and 17 years. Exclusion was primarily due to unavailability for follow-up. Administrative medical data linkage was available for 100% of the analytic sample.

### Exposure

Adolescents reported past-year cannabis use at ages 12, 13, 15, and 17 years. Responses ranged from “I didn’t” (0) to “every day” (6) (eTable 1 in Supplement 1).

### Outcomes

The ISQ provided access to QLSCD data linked to anonymized administrative medical care databases, including physician claims (Regie de l’assurance maladie du Québec), inpatient discharge records (Maintenance et exploitation des données pour l’étude de la cientèle hospitalière), and emergency department admissions (Banque de données communes des urgences [BDCU]), providing primary and secondary *International Statistical Classification of Diseases, Ninth Revision* (*ICD-9*) and *ICD-10* diagnoses from birth (1997-1998) to age 23 years (2021) (BDCU available from 16 years of age) (eTable 2 in Supplement 1). We created dichotomous indicators of any medical care use between ages 18 and 23 years. Main outcomes were any mental disorder, including common mental disorders (depressive, anxiety, or adjustment disorders), severe mental disorders (bipolar or schizophrenia spectrum and other psychotic disorders), and substance-related disorders (alcohol-, cannabis-, and other drug-related disorders), and any physical health condition, encompassing diseases of the respiratory system, injuries, and poisoning, and other physical diseases. Secondary outcomes included common mental disorders, substance-related disorders, suicide-related behaviors (including suicide attempt and suicidal ideation), diseases of the respiratory system, injuries and poisoning, and other physical diseases. The other physical diseases category included conditions (eg, gastrointestinal tract, neurological, infectious, and cardiovascular) not captured in the prior groups but identified in the literature as potentially associated with cannabis use.^6^ A complete list of *ICD* codes is provided in eTable 2 in Supplement 1.

Outcome selection was guided by substantive knowledge (eTable 2 in Supplement 1) and used a minimum of 10 cases per exposure stratum to ensure a stable model estimation and to comply with ISQ identity-protection guidelines. This threshold was applied only to define outcome-specific analytic samples and did not reflect the number of parameters estimated in outcome models. Linkage to administrative medical care databases was performed using participants’ unique health insurance numbers.

### Covariates

We identified 32 preexposure individual, family, and community-level confounders using a directed acyclic graph informed by substantive expertise (eAppendix 1 and eFigure 1 in Supplement 1). Table 1^28,29,30,31,32,33,34,35,36,37,38,39,40,41,42,43,44,45^ details the confounders extracted from the QLSCD, including the survey waves used for their assessment and descriptions of each variable. These confounders, measured via validated instruments, capture a wide range of early-life vulnerabilities from birth to younger than 12 years of age. Additional data from administrative medical care databases included sex (male or female, as recorded at birth in health records, which does not include gender identity) and utilization of medical care before age 12 years for any mental disorder, neurodevelopmental disorders (including disabilities, disorders of psychological development, attention-deficit/hyperactivity disorder, stereotyped movement disorders, and tic disorders), disturbances of conduct and emotions occurring in childhood or adolescence, and any physical health condition. Management of the administrative medical care databases followed the same approach outlined for the outcomes (eTable 2 in Supplement 1). Self-reported use of tobacco (past 30 days, scored from 1 [“never used”] to 5 [“every day”]) and alcohol (past 12 months, scored from 1 [“I didn’t use] to 7 [“every day”]) were assessed at ages 12, 13, 15, and 17 years. Parental alcohol use was coded on an ordinal frequency scale: 7, every day; 6, 4 to 6 times per week; 5, 2 to 3 times per week; 4, once a week; and 1, less than once a month. Socioeconomic status is expressed as a *z* score from a composite deprivation index.

**Table 1.** Study Confounders Extracted From the Québec Longitudinal Study of Child Development (QLSCD)

Confounder	Survey wave (child age, y)^a^										Brief description^b^
	0.5	1.5	2.5	3.5	4.0	5.0	6.0	7.0	8.0	10.0	
Maternal age	Yes	No	No	No	No	No	No	No	No	No	Maternal age when child was ~ 5 mo old: ≤24 y, 25 to 29 y, 30 to 34 y, and ≥35 y
Parental ethno-cultural origins^c^	Yes	No	No	No	No	No	No	No	No	No	Parents Canadian-born or born outside Canada
Family type	Yes	No	No	No	No	No	No	No	No	No	Intact, 2-parent families or blended/single-parent families
Household socioeconomic status	Yes	Yes	Yes	Yes	No	Yes	Yes	Yes	Yes	Yes	NLSCY,^28^ composite score, mean (SD): 0 (3) of parental education, occupational status, income
Neighborhood conflict^d^	Yes	Yes	No	No	Yes	No	Yes	No	Yes	Yes	NQ,^29^ PMK-reported perception of neighborhood unsafety and lack of social cohesion (n = 5)
Maternal prenatal substance use^e^	Yes	No	No	No	No	No	No	No	No	No	NLSCY and DIS,^30,31^ maternal-reported use of tobacco, alcohol, or illegal drugs during pregnancy
Parental substance use^f^	Yes	Yes	Yes	Yes	No	No	No	No	No	No	NLSCY and DIS,^30,31^ parental-reported use of tobacco, alcohol, or illegal drugs during the past 12 mo
Maternal depression	Yes	Yes	No	Yes	No	Yes	No	Yes	No	Yes	CES-D,^32^ maternal self-report of depressive symptoms (n = 6)
Family dysfunction^g^^,^^h^	Yes	Yes	No	No	No	No	Yes	No	No	No	CMHFFS,^33^ PMK-reported family dynamics, emotional connection, and behavioral guidance (n ~ 6)
Positive parenting practices^g^	No	No	Yes	Yes	Yes	Yes	Yes	No	Yes	Yes	NLSCY and PPS,^30,34^ PMK-reported supportive interactions and nurturing care (n ~ 3)
Coercive parenting practices^g^	No	No	Yes	Yes	Yes	Yes	Yes	No	Yes	Yes	NLSCY and PPS,^30,34^ PMK-reported negative and hostile interactions (n ~ 3)
Consequent parenting practices^g^	No	No	Yes	Yes	Yes	Yes	Yes	No	Yes	Yes	NLSCY and PPS,^30,34^ PMK-reported application of consistent rules for similar behaviors (n ~ 4)
Parental monitoring^g^	No	No	No	No	No	No	No	No	No	Yes	PMS,^35^ PMK-reported knowledge of child’s peer network, communication, and oversight (n = 5)
Verbal skills	No	No	No	Yes	No	Yes	Yes	No	No	Yes	PPVT-R,^36^ age-corrected and standardized scores, mean (SD): 100 (15)
Internalizing behaviors^g^	No	No	No	No	No	No	Yes	Yes	Yes	Yes	SBQ,^37,38^ teacher-rated anxiety (n = 4), emotional distress (n ~ 5), and withdrawal (n ~ 4)
Externalizing behaviors^g^	No	No	No	No	No	No	Yes	Yes	Yes	Yes	SBQ,^37,38^ teacher-rated aggression (n = 10), opposition (n = 4), and inattention/hyperactivity (n = 9)
Social skills^g^	No	No	No	No	No	No	Yes	Yes	Yes	Yes	EDI,^39^ teacher-rated sociability (n ~ 9) and responsibility (n ~ 6)
Victimization^g^	No	No	No	No	No	No	Yes	Yes	Yes	Yes	SRVS,^40^ self-reported verbal, physical, and relational peer victimization (n ~ 5)
Academic performance^i^	No	No	No	No	No	No	No	Yes	Yes	Yes	NLSCY,^41^ teacher-rated overall academic performance (n = 1)
Depressive symptoms^g^	No	No	No	No	No	No	No	No	No	Yes	CDI,^42^ self-reported depressive symptoms (n = 8)
Deviant behaviors^g^	No	No	No	No	No	No	No	No	No	Yes	NLSCY and QNTS,^43,44^ self-reported risk-taking and rule-breaking behaviors (n = 9)

Abbreviations: CDI, Child Depression Inventory; CES-D, Center for Epidemiologic Studies Depression Scale; CMHFFS, Chedoke-McMaster Hospital Family Functioning Scale; DIS, Diagnostic Interview Schedule; EDI, Early Development Instrument; NLSCY, National Longitudinal Survey of Children and Youth (Canada); NQ, Neighborhood Questionnaire; PMK, person most knowledgeable of the child (in QLSCD, it is usually the biological mother^45^); PMS, Parental Monitoring Scale; PPS, Parent Practices Scale; PPVT-R, Peabody Picture Vocabulary Test-Revised; QNTS, Quebec Newborn Twin Study; SBQ, Social Behavior Questionnaire; SRVS, Self-Report Victimization Scale.

^a^ Yes or No were used to indicate whether each confounder was included at each wave. The mean of variables measured at different time points was obtained.

^b^ “n” represents the number of items, with “~” signaling the minimum number of items used across survey waves to ensure consistency in derived variable construction. The change in the number of items reflects pragmatic adjustments (eg, shorter scales), ensuring consistent construct measurement across waves through standardization and use of minimum common items.

^c^ Refers to 2 separate variables, 1 for each parent.

^d^ Scored on a scale of 1 to 4, with higher scores indicating greater levels of neighborhood conflict.

^e^ Refers to 3 variables, 1 per substance: alcohol (0 to 6 Likert-type scale: “never” to “every day”), tobacco (yes or no), and illegal drugs (yes or no), with 1 question per substance.

^f^ Refers to 6 separate variables, 1 per substance: alcohol (0 to 7 Likert-type scale: “never” to “every day”), tobacco (3-point Likert-type scale: “not really,” “used occasionally,” and “used every day”), and illegal drugs (yes or no), with 1 question per substance. Parents were coded with a positive score for illegal drug use if they ever answered yes.

^g^ Scores were standardized on a scale of 0 to 10, with higher scores indicating greater levels of the measured construct.

^h^ Scores were reversed from the original scale so that higher scores reflect greater family dysfunction.

^i^ Scores were measured on a scale of 1 to 5, with higher scores reflecting better academic performance.

### Statistical Analysis

Group-based trajectory modeling (GBTM), a finite mixture approach,^46^ was used to identify subpopulations with distinct patterns of cannabis use across adolescence. Unlike variable-centered approaches that model continuous variation on a single dimension (eg, either cumulative use, age of onset, or intensity of use),^47^ GBTM uses a person-centered approach that identifies qualitatively distinct usage profiles that reflect real-world heterogeneity in timing and intensity, enabling more precise identification of at-risk subgroups.

GBTM used the maximum likelihood estimation, retaining cases with at least 1 data point. Nonrandom attrition was assumed to occur at a constant rate within each group. Males and individuals from households of lower socioeconomic status were more likely to have missing follow-up cannabis data within the study sample; thus, parameters were estimated using a conditional estimator, with these covariates used to model group membership and dropout probability. Count and zero-inflated count distribution models were tested for the cannabis data. Time was measured in years (12-17) in the statistical model. The mean ages at each wave were 12.1, 13.1, 15.1, and 17.2 years, with SDs of 0.2, indicating minimal variability in time intervals across individuals.

An automated algorithm optimized the bayesian information criterion to select the optimal number of trajectories (1-5 groups) and the best polynomial order (constant, linear, or quadratic). We evaluated 40 combinations. Final model selection followed the Nagin recommended criteria: highest bayesian information criterion and probability correct model, mean posterior probabilities at least 0.7, odds of correct classification 5.0 or higher, entropy 0.700 or higher, and substantive interpretability.^46^ To confirm that the class solution did not converge to a local maximum of the maximum likelihood distribution, the model was rerun with 100 sets of randomly generated starting values.^48^

For missing covariate data, multiple imputation through fully conditional specification was used to handle arbitrary missing value patterns.^49^ We generated 20 imputed datasets with 30 burn-in iterations each. To investigate the association between patterns of cannabis use and medical care utilization, we fitted unadjusted and overlap-weighted logistic regression models, reporting odds ratios (ORs) and their 95% CIs. In overlap-weighted models, weights were derived from a multinomial propensity score model including 32 early-life confounders. These weights strongly balanced the covariate distributions across exposure groups without including them directly in the outcome model, thereby mitigating confounding while avoiding overfitting.^50^ The overlap-weighted analysis targeted individuals with similar probabilities of belonging to different cannabis use trajectories (ie, the population in clinical equipoise).^50^ Confounders were assessed for multicollinearity by ensuring a variance inflation factor lower than 10 in the exposure model. Covariate balance after weighting was assessed using standardized mean differences (SMDs), with values lower than 0.1 indicating adequate balance.^50^ Inverse-probability weights were applied in all estimations to account for differences between the study sample and the complete cohort based on sex and household socioeconomic status.

Considering sex-dependent differences in cannabis use responses,^51^ as well as the complex associations among different types of substance use during adolescence—in which evidence suggests cannabis use sometimes precedes tobacco or alcohol initiation—^52^ we examined whether sex or adolescent tobacco or alcohol use modified the associations between patterns of adolescent cannabis use and medical care utilization for any mental disorder or any physical health condition using interaction terms.

All analyses were conducted from November 2023 to February 2025 in Stata 18.0 (Stata Corp LLC), with the Stata code used for the analysis available online.^53^ Significance thresholds were set at *P* < .05, and all tests were 2-tailed.

## Results

Among 1591 participants (818 female [51.4%], 773 male [48.6%]) followed from birth to 23 years of age, the mean (SD) age at first cannabis exposure assessment was 12.1 (0.3) years, with 348 participants (21.9%) born to mothers younger than 24 years, and 298 participants (18.7%) living in step- or single-parent households during early childhood (Table 2).

**Table 2.** Characteristics of the Participants Included in the Study

Characteristic	Participants, No. (%)				
	Trajectories of cannabis use during adolescence				
	No use	Late-onset use	Early-onset and frequent use	Total	*P* value^a^
Total	948 (59.6)	318 (20.0)	325 (20.4)	1591 (100)	NA
Sex					
Female	467 (49.3)	164 (51.6)	187 (57.5)	818 (51.4)	.04
Male	481 (50.7)	154 (48.4)	138 (42.5)	773 (48.6)	
Internalizing behaviors, mean (SD)^b^	2.4 (1.5)	2.1 (1.3)	2.1 (1.2)	2.3 (1.4)	.001
Depressive symptoms, mean (SD)^b^	1.4 (1.4)	1.4 (1.5)	1.4 (1.4)	1.4 (1.4)	.52
Externalizing behaviors, mean (SD)^b^	1.7 (1.8)	1.8 (1.8)	1.9 (1.7)	1.8 (1.8)	.10
Deviant behaviors, mean (SD)^b^	0.7 (1.0)	0.9 (1.2)	1.0 (1.1)	0.8 (1.0)	<.001
Verbal skills, mean (SD)^c^	98.5 (13.4)	101.6 (11.2)	100.2 (10.2)	99.5 (12.4)	<.001
Academic performance, mean (SD)^d^	3.1 (1.4)	2.8 (1.2)	3.2 (1.3)	3.0 (1.3)	.001
Social skills, mean (SD)^b^	7.7 (1.4)	7.8 (1.3)	7.5 (1.2)	7.7 (1.4)	.02
Victimization, mean (SD) ^b^	3.3 (1.8)	3.5 (1.9)	3.8 (1.8)	3.4 (1.9)	<.001
Any mental and behavioral disorder^e^^,^^f^	180 (19.0)	40 (12.6)	55 (16.9)	275 (17.3)	.03
Any physical condition^g^	460 (48.5)	155 (48.7)	140 (43.1)	755 (47.5)	.24
Lifetime (adolescence) tobacco use	176 (23.6)	208 (65.4)	297 (95.8)	681 (49.6)	<.001
Lifetime alcohol use	645 (79.6)	314 (98.7)	318 (98.8)	1277 (88.1)	<.001
Cannabis use during the past 12 mo, mean (SD)^h^	0.0 (0.0)	2.1 (1.3)	3.3 (1.9)	1.2 (1.7)	<.001
Maternal age, y					
<24	180 (19.0)	67 (21.1)	101 (31.1)	348 (21.9)	<.001
25-29	324 (34.2)	90 (28.3)	91 (28.0)	505 (31.7)	
30-34	314 (33.1)	108 (34.0)	99 (30.5)	521 (32.7)	
≥35	130 (13.7)	53 (16.7)	34 (10.5)	217 (13.6)	
Canadian-born mother	586 (62.1)	213 (67.4)	234 (72.9)	1033 (65.3)	.001
Canadian-born father	565 (64.9)	214 (72.1)	206 (70.5)	985 (67.5)	.03
Step- or single-parent family	175 (18.5)	46 (14.5)	75 (23.1)	296 (18.7)	.02
Household socioeconomic status, mean (SD), *z* score	−0.0 (0.9)	0.1 (0.9)	−0.3 (0.9)	−0.0 (0.9)	<.001
Neighborhood conflict^i^	1.8 (0.6)	1.8 (0.6)	1.8 (0.6)	1.8 (0.6)	.51
Family functioning^b^	1.7 (1.4)	1.6 (1.4)	1.8 (1.5)	1.7 (1.5)	.23
Positive parenting practices, mean (SD)^b^	6.3 (0.8)	6.1 (0.8)	6.3 (0.8)	6.2 (0.8)	.02
Coercive parenting practices, mean (SD)^b^	2.6 (0.9)	2.6 (0.8)	2.7 (0.9)	2.6 (0.9)	.19
Consequent parenting practices, mean (SD)^b^	7.1 (1.2)	7.1 (1.2)	6.9 (1.2)	7.0 (1.2)	.03
Parental monitoring at age 10, mean (SD)^b^	8.9 (1.0)	9.0 (0.8)	8.8 (0.9)	8.9 (1.0)	.04
Maternal depression, mean (SD)^b^	1.4 (1.1)	1.3 (1.1)	1.4 (1.0)	1.4 (1.1)	.14
Maternal prenatal tobacco use	204 (21.6)	67 (21.2)	118 (36.5)	389 (24.6)	<.001
Maternal prenatal alcohol use, mean (SD)^j^	0.5 (0.8)	0.5 (0.8)	0.6 (0.8)	0.5 (0.8)	.48
Maternal prenatal drug use^k^	NA	NA	NA	NA	.08
Maternal tobacco use					
Not really	727 (76.8)	239 (75.2)	195 (60.0)	1161 (73.0)	<.001
Used occasionally	33 (3.5)	9 (2.8)	14 (4.3)	56 (3.5)	
Used everyday	187 (19.7)	70 (22.0)	116 (35.7)	373 (23.5)	
Maternal alcohol use mean (SD)^j^	1.6 (1.5)	1.8 (1.6)	2.1 (1.6)	1.7 (1.6)	<.001
Maternal drug use	34 (3.6)	12 (3.8)	19 (5.8)	65 (4.1)	.20
Paternal tobacco use					
Not really	627 (71.6)	216 (71.1)	161 (54.4)	1004 (68.0)	<.001
Used occasionally	24 (2.7)	13 (4.3)	10 (3.4)	47 (3.2)	
Used everyday	225 (25.7)	75 (24.7)	125 (42.2)	425 (28.8)	
Paternal alcohol use, mean (SD) ^j^	3.1 (1.9)	3.6 (1.9)	3.6 (1.9)	3.3 (1.9)	<.001
Paternal drug use	56 (6.4)	23 (7.6)	39 (13.2)	118 (8.0)	.001

Abbreviation: NA, not available.

^a^ *P* values were obtained from a pooled *t* test for continuous variables and Pearson χ^2^ for categorical variables.

^b^ Scores were standardized on a scale of 0 to 10, with higher scores indicating greater levels of the measured construct.

^c^ Standardized score mean, 100 with SD 15.

^d^ Scores were measured on a scale of 1 to 5, with higher scores reflecting better academic performance.

^e^ Any medical care use before 12 years of age for a diagnosed common, severe, substance-related, neurodevelopmental, or conduct/emotions disorder.

^f^ Data for medical care use were rounded to the nearest multiple of 5 to prevent identification of individuals when Institut de la Statistique du Québec (ISQ) data were compiled and compared with other publicly available statistics.

^g^ Any medical care use before 12 years of age for a diagnosed respiratory disease, injury or poisoning, or other physical disease.

^h^ Responses ranged from 0, “I didn’t” to 6, “every day.”

^i^ Scored on a scale of 1 to 4, with higher scores indicating greater levels of neighborhood conflict.

^j^ Likert-type scale ranging from 0, “never,” to 7, “every day.”

^k^ Data for maternal prenatal drug use was not shown to comply with ISQ participant identity-protection guidelines, as 1 of the exposure strata had fewer than 5 observations.

### Patterns of Adolescent Cannabis Use

**Figure 1.**
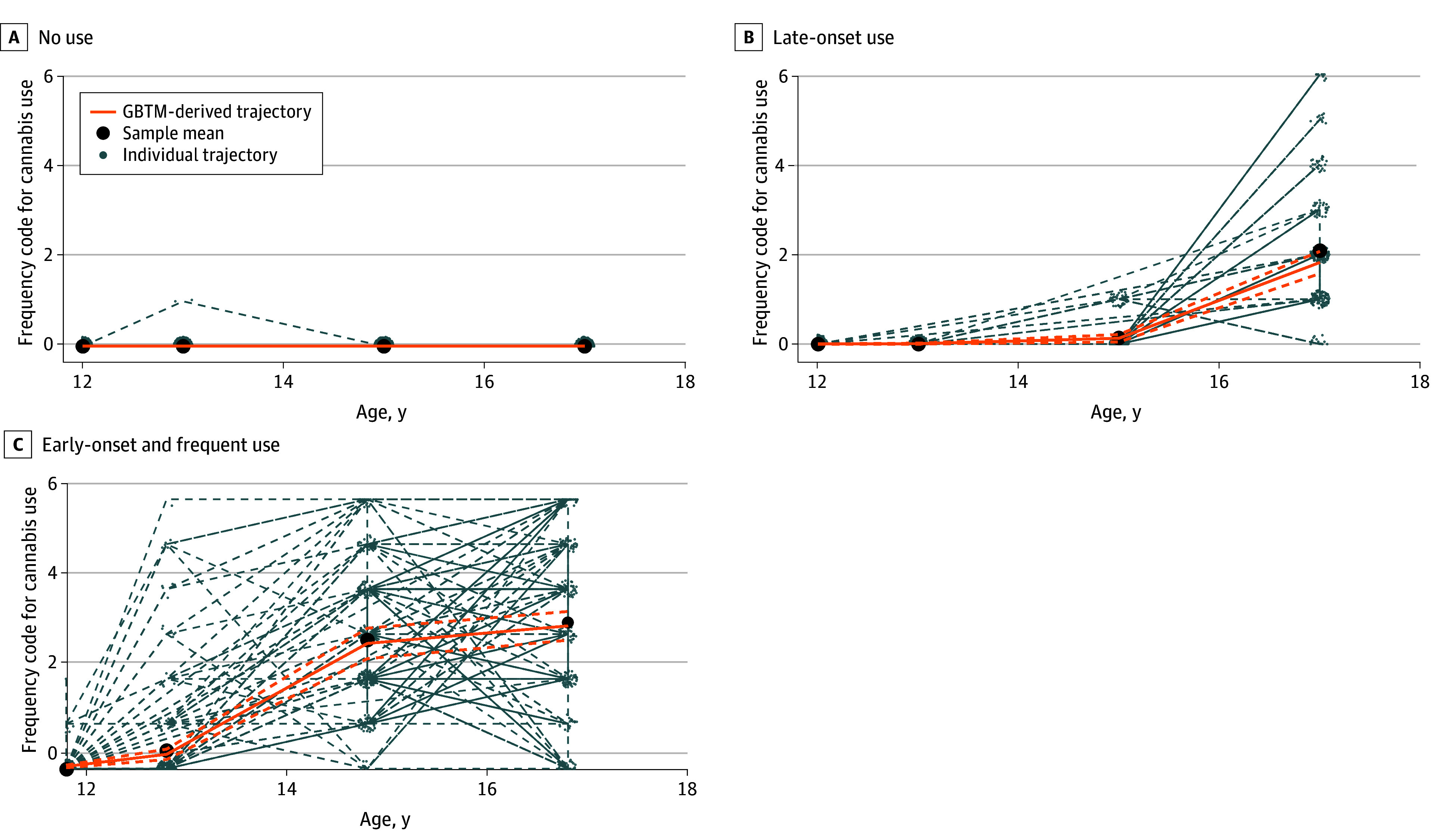
Patterns of Cannabis Use During Adolescence Orange dashed lines indicate 95% confidence intervals of the respective trajectory. Frequency codes for cannabis use during the past 12 months were categorized as 0, “I didn’t use”; 1, “just once”; 2, “less than once a month or occasionally”; 3,”about once a month”; 4, “during the weekend or 1 to 2 times a week”; 5, “more than 3 times a week”; and 6, “every day.” GBTM indicates group-based trajectory modeling.

We identified 3 patterns of adolescent cannabis use using GBTM with a count distribution model (Figure 1). The estimated parameters for each trajectory are presented in eTable 3 in Supplement 1. The identified groups were (1) individuals who never used cannabis during adolescence (nonuse, 948 [59.6%]); (2) individuals who started using cannabis after 15 years of age and by age 17 years used cannabis less than once a month (late-onset use, 318 [20.0%]); and (3) individuals who began using cannabis before 15 years of age and used cannabis at least once per month (early-onset and frequent use, 325 [20.4%]). The 3-group solution had a probability correct model of 0.99. All groups identified by GBTM had a mean posterior probability of at least 88.1%, and odds of correct classification equal or higher than 5.0, and an entropy of 0.719, indicating good model fit.^46^ After rerunning the model with different sets of starting values, the model consistently converged and led to the same maximum likelihood estimations 91.0% of the time.

### Child and Family Characteristics Across Patterns of Adolescent Cannabis Use

Individuals with early-onset and frequent cannabis use displayed more deviant behaviors (mean [SD], 1.0 [1.1] vs 0.7 [1.0] for nonuse and 0.9 [1.2] for late-onset use), experienced greater peer victimization (mean [SD], 3.8 (1.8) vs 3.3 [1.8] for nonuse and 3.5 [1.9] for late-onset use), and were notably more likely to have used tobacco themselves (297 [95.8%] vs 176 [23.6%] for nonuse and 208 [65.4%] for late-onset use) (Table 2). In addition, individuals with early-onset and frequent use were more likely to be female (187 [57.5%] compared with 138 [42.5%] male). In contrast, individuals with no use exhibited somewhat higher internalizing behaviors (mean [SD], 2.4 [1.5] vs 2.1 [1.3] for late-onset use and 2.1 [1.2] for early-onset and frequent use). Individuals with early-onset and frequent use had more maternal prenatal tobacco exposure (118 [36.5%] vs 204 [21.6%] for nonuse and 67 [21.2%] for late-onset use), higher daily paternal tobacco use (125 [42.2%] vs 225 [25.7%] for nonuse and 75 [24.7%]) for late-onset use) and drug use (39 [13.2%] vs 56 [6.4%] for nonuse and 23 [7.6%] for late-onset use), and increased parental alcohol use (mean [SD] score, 3.6 [1.9] vs 3.1 [1.9] for nonuse and 3.6 [1.9] for late-onset use) as well as overall lower household socioeconomic status (mean [SD] score, −0.3 [0.9] vs −0.0 [0.9] for nonuse and 0.1 [0.9] for late-onset use).

### Patterns of Adolescent Cannabis Use and Medical Care Utilization for Mental and Physical Health Conditions

#### Primary Outcomes

**Figure 2.**
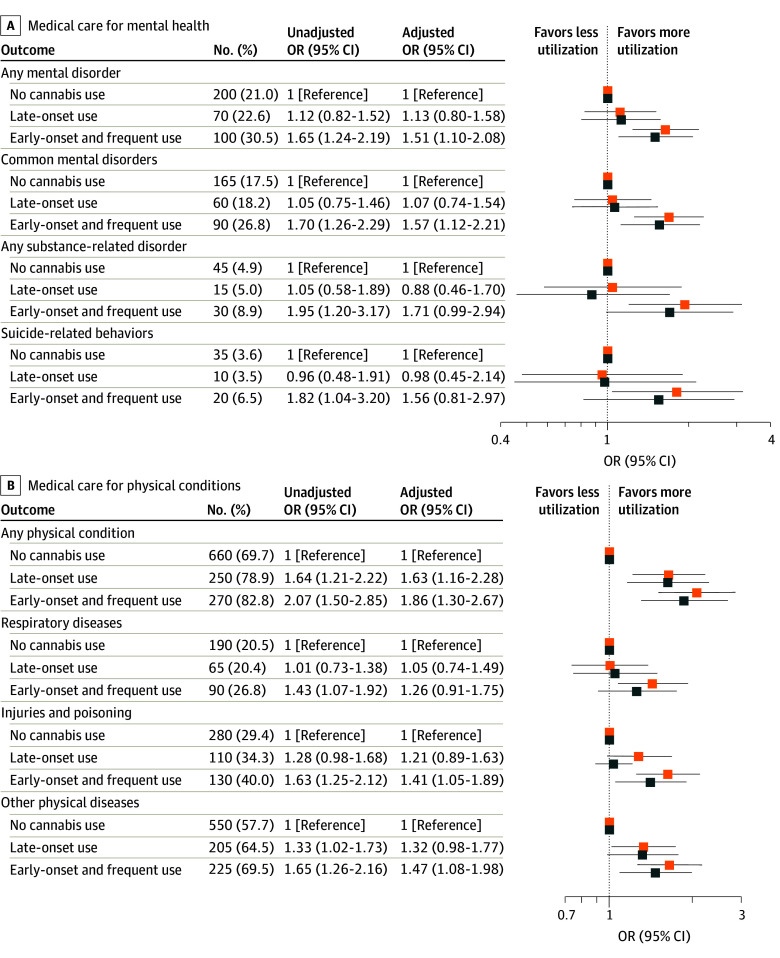
Patterns of Cannabis Use During Adolescence and Medical Care Utilization for Mental Health and Physical Health Age 18 to 23 Years Orange squares represent unadjusted odds ratios (ORs), and blue squares represent adjusted ORs estimated using overlap weighting to address confounder imbalance. Inverse probability weighting was applied to account for sampling differences between the study sample and the complete cohort. Data for medical care use were rounded to the nearest multiple of 5 in accordance with confidentiality requirements of the Institut de la statistique du Québec and compared with publicly available statistics.

**Figure 3.**
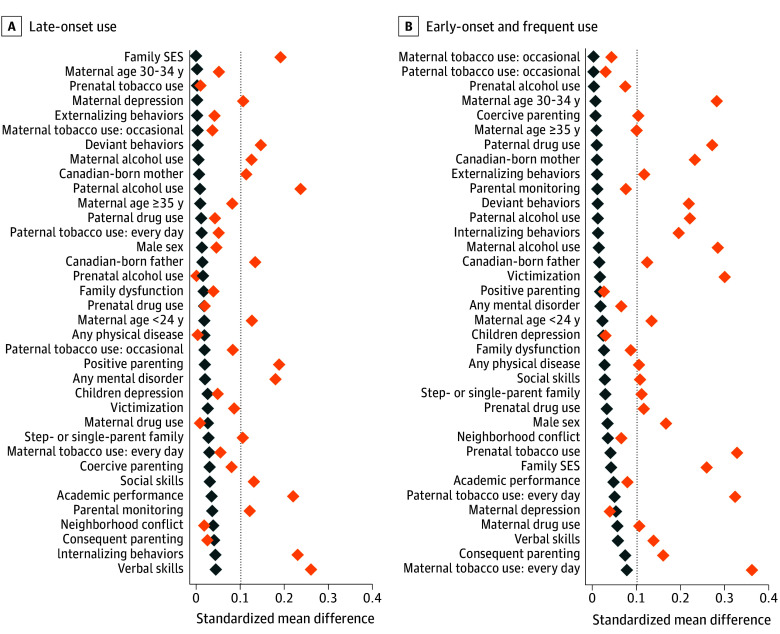
Confounder Balance in the Study Sample Absolute (pairwise) standardized mean differences for comparisons between the groups exposed to cannabis use vs the nonexposed group. Dashed lines denote the commonly accepted threshold of 0.1 for standardized mean differences indicating adequate confounder balance; orange diamonds, unweighted estimates; blue diamonds, overlap-weighted estimates (after applying overlap weights to balance confounders). SES indicates socioeconomic status.

In unadjusted analyses, individuals with early-onset and frequent use had higher odds of receiving medical care for any mental disorder (unadjusted OR [uOR], 1.65 [95% CI, 1.24-2.19]) and any physical condition (uOR, 2.07 [95% CI, 1.50-2.85]) compared with individuals with no use (Figure 2). After balancing the distribution of confounders using overlap weighting (Figure 3), adjusted estimates were slightly attenuated but remained increased for having any mental disorder (adjusted OR [aOR], 1.51 [95% CI, 1.10-2.08]) and any physical condition (aOR, 1.86 [95% CI, 1.30-2.67]) (Figure 2). For individuals with late-onset use, the aOR for having any mental disorder was 1.13 (95% CI, 0.80-1.58), and for having any physical condition, the aOR was 1.63 (95% CI, 1.16-2.28), indicating an association only for the latter.

#### Secondary Outcomes

For specific mental disorders (Figure 2A), individuals with early-onset and frequent use had significantly increased odds of receiving medical care for common mental disorders (uOR, 1.70 [95% CI, 1.26-2.29]; aOR, 1.57 [95% CI, 1.12-2.21]), but not for substance-related disorders (uOR, 1.95 [95% CI, 1.20-3.17]; aOR, 1.71 [95% CI, 0.99-2.94]) or suicide-related behaviors (uOR, 1.82 [95% CI, 1.04-3.20]; aOR, 1.56 [95% CI, 0.81-2.97]). For specific physical conditions (Figure 2B), individuals with early-onset and frequent use showed higher unadjusted odds of having respiratory disease (uOR, 1.43 [95% CI, 1.07-1.92]), injury and poisoning (uOR, 1.63 [95% CI, 1.25-2.12]), and other physical disease (uOR, 1.65 [95% CI, 1.26-2.16]). After overlap weighting, odds remained significantly increased for injury and poisoning (aOR, 1.41 [95% CI, 1.05-1.89]) and other physical disease (aOR, 1.47 [95% CI, 1.08-1.98]) but not for respiratory disease (aOR, 1.26 [95% CI, 0.91-1.75]). Among individuals with late-onset use, adjusted odds for having injury and poisoning (aOR, 1.21 [95% CI, 0.89-1.63]) and other physical disease (aOR, 1.32 [95% CI, 0.98-1.77]) were not statistically significant.

Results from the nonimputed sample were consistent across key outcomes (eTable 4, eFigure 2 in Supplement 1). No statistically significant interactions by sex or adolescent tobacco or alcohol use and patterns of adolescent cannabis use were found (eAppendix 2 and eTable 5 in Supplement 1).

## Discussion

In this longitudinal birth cohort study linking adolescent self-reports of cannabis use to objective medical care databases from Québec, Canada, we found that adolescents who initiated cannabis use early and continued consistently accessed more medical care for both mental and physical conditions in young adulthood, compared with their peers who did not use cannabis. These associations persisted even after rigorous adjustment for a wide range of preexposure confounding factors, including early individual, familial, and community-based vulnerabilities.

Our findings extend previous research showing that early-onset and frequent cannabis use was associated with higher odds of conditions such as common mental disorders^12,13,14,15,16,17^ and substance-related disorders^12,13,14,15,16,18,19,20,21,22,23,24^ in adulthood. Beyond mental health, participants with early-onset and frequent cannabis use showed increased odds of medical care use for physical health conditions, notably injuries and poisonings. These increased risks may reflect neurocognitive and behavioral changes associated with early-onset and frequent cannabis exposure,^54,55,56,57,58^ including acute intoxication symptoms and withdrawal-related responses, as well as the broader availability of potent cannabis products that can lead to accidental overdose.^56^ Although we did not observe consistent associations with chronic respiratory diseases, it is possible that these outcomes will emerge with longer follow-up, given that many physical diseases have longer latency periods.^6,59^ Lifestyle factors associated with early cannabis use, such as poor diet and insufficient sleep,^16,25^ may compound these risks over time. These findings underscore the importance of ongoing, systematic monitoring of youth cannabis use and associated health outcomes, especially given the increased accessibility, diversity, and potency of cannabis products that continue to expand in many jurisdictions in North America and internationally.^60^

Unlike most prior investigations relying on self-reported health outcomes,^13,15,18,21,22,23,24^ our study leveraged data from comprehensive medical registries, thereby capturing clinically significant conditions that required medical intervention. While previous studies suggested that both early and late adolescent cannabis use could lead to poor health outcomes,^12,13,14,15,16,18,20,21,22,23,24^ we found that individuals with late-onset use did not differ significantly from those with nonuse in receipt of mental health–related medical care, yet they did exhibit higher odds of physical health conditions (including injuries and poisoning). Even after accounting for a wide range of early-life and family-level confounders, early-onset and frequent use remained associated with later medical care utilization, raising the possibility that cannabis exposure during a period of rapid development may be associated with adverse outcomes beyond what can be fully explained by preexisting vulnerabilities. This finding highlights the critical role of early and frequent cannabis use in shaping long-term health trajectories, suggesting that efforts to delay initiation or reduce frequency of early use may help mitigate potential long-term harms.^61,62,63,64^ However, the increased risk of physical conditions observed even among individuals with late-onset use reinforces the importance of monitoring this group to better characterize their long-term health care needs.

The absence of significant associations in some of our adjusted models, such as those for suicide-related behaviors, may partly reflect limited statistical power and relatively low base rates of such events. However, it is also plausible that cannabis use initiated in later adolescence has weaker or no association with certain outcomes, consistent with developmental literature on early exposure risk.^3,4,12,15^ Nonetheless, the patterns observed warrant continued monitoring, given the potential severity of these outcomes. Moreover, no significant interactions by sex or by co-occurring adolescent tobacco or alcohol use were found, indicating that the adverse consequences of early-onset and frequent cannabis use on medical care utilization are broadly similar across these subgroups. Longer-term studies are needed to investigate whether this pattern is sustained or evolves into adulthood. Future studies should also investigate potential mechanisms explaining these associations, including assessing the role of continuing cannabis use in the postadolescent years.

Finally, we found that individuals with early cannabis use differed substantially from those with late use and those with no cannabis use in their exposure to early-life risk factors, including parental substance use,^22,24^ which is known to influence mental health and substance use trajectories in offspring.^22^ Such findings underscore the importance of also addressing underlying vulnerabilities in prevention strategies and policy formation. These early-life determinants are not only confounders but also upstream factors associated with adolescent cannabis exposure.^22,24^ Population-level interventions that support families with young children may represent a viable path toward reducing cannabis-associated harms across development.^62,63^

### Limitations

Several limitations should be noted. First, despite rigorous adjustments for various confounding factors, residual confounding remained possible given unmeasured genetic predispositions, which may have influenced both cannabis use and health outcomes. Second, selective attrition was more prevalent among certain subgroups, particularly male participants and participants from lower socioeconomic backgrounds, which may have influenced the distribution of confounders in ways that partly explained the unexpected childhood profiles (eg, no cannabis use showing relatively higher internalizing behaviors. Third, although we used medical records to improve the validity of participant outcome data, some diagnostic codes (particularly for physical injuries and poisoning) were not routinely or consistently used, potentially leading to an underestimation of the true magnitude of associations. Fourth, our data did not capture subclinical symptoms or functional impairments that did not result in medical visits, potentially underestimating the broader impact of adolescent cannabis use on daily functioning and health trajectories. Fifth, adolescents with earlier or more frequent cannabis use may avoid medical care services due to stigma or other risk factors, thus reducing detection of relevant conditions. Sixth, self-reported measures of cannabis use may be subject to underreporting or recall bias. In addition, the generalizability of these findings may be constrained by the cohort’s geographic and temporal context—participants were adolescents prior to the 2018 legalization of nonmedical cannabis in Canada—and their patterns of use and associated risk may not reflect those observed in postlegalization cohorts. Moreover, increasing product potency, changing usage patterns, and differences in accessibility of medical services may further limit applicability to current populations.

## Conclusions

In this population-based birth cohort study, early-onset and frequent cannabis use during adolescence was associated with increased medical care utilization for both mental and physical health conditions in young adulthood. Participants with late-onset cannabis use also showed increased utilization of medical care for specific physical conditions, highlighting risks across onset patterns. By integrating repeated adolescent self-reports with objective administrative medical records, and accounting for early-life confounders, this study offers insight into cannabis-associated medical care burdens in a universal health care setting. As access to high-potency cannabis expands, efforts to delay initiation and reduce frequency of adolescent use should remain a public health priority. Identifying adolescents with early risk profiles and attending to psychosocial and familial factors may help inform strategies to address future health care needs.

## Notes

Supplement 1. **eTable 1.** Distribution of cannabis use during adolescence in the response sample **eTable 2.** Diagnostic definitions and codes for mental, neurodevelopmental, and physical health conditions using administrative medical records **eAppendix 1.** Selection of pre-exposure individual, family, and community-level confounders **eFigure 1.** Directed acyclic graph – developmental pathways to adolescent cannabis use and utilization of medical care **eTable 3.** Final parameter estimates from the group-based trajectory model **eTable 4.** Patterns of cannabis use during adolescence and medical care utilization for mental or physical health conditions between ages 18 to 23 in the response sample **eFigure 2.** Confounder balance in the response sample **eAppendix 2.** Interaction analyses for sex, tobacco use, and alcohol use **eTable 5.** Stratified analyses by sex (adjusted estimations)

Supplement 2.
Data Sharing Statement
