# Supplementary material for: Health Service Use Among Young Adults With a History of Adolescent Cannabis Use
Source: JAMA Netw Open. 2025 Oct 28;8(10):e2539977. doi: 10.1001/jamanetworkopen.2025.39977 (PMC12569714; doi:10.1001/jamanetworkopen.2025.39977)
Supplement: Supplement 1. — eTable 1. Distribution of cannabis use during adolescence in the response sample eTable 2. Diagnostic definitions and codes for mental, neurodevelopmental, and physical health conditions using administrative medical records eAppendix 1. Selection of pre-exposure individual, family, and community-level confounders eFigure 1. Directed acyclic graph – developmental pathways to adolescent cannabis use and utilization of medical care eTable 3. Final parameter estimates from the group-based trajectory model eTable 4. Patterns of cannabis use during adolescence and medical care utilization for mental or physical health conditions between ages 18 to 23 in the response sample eFigure 2. Confounder balance in the response sample eAppendix 2. Interaction analyses for sex, tobacco use, and alcohol use eTable 5. Stratified analyses by sex (adjusted estimations) [file jamanetwopen-e2539977-s001.pdf]

Supplementary Online Content

Martínez P, Chadi N, Castellanos-Ryan N, et al. Health service use among young adults with a history of adolescent cannabis use. *JAMA Netw Open*. 2025;8(10):e2539977. doi:10.1001/jamanetworkopen.2025.39977

eTable 1. Distribution of cannabis use during adolescence in the response sample

eTable 2. Diagnostic definitions and codes for mental, neurodevelopmental, and physical health conditions using administrative medical records

eAppenix 1. Selection of pre-exposure individual, family, and community-level confounders

eFigure 1. Directed acyclic graph – developmental pathways to adolescent cannabis use and utilization of medical care

eTable 3. Final parameter estimates from the group-based trajectory model

eTable 4. Patterns of cannabis use during adolescence and medical care utilization for mental or physical health conditions between ages 18 to 23 in the response sample

eFigure 2. Confounder balance in the response sample

eAppendix 2. interaction analyses for sex, tobacco use, and alcohol use

eTable 5. Stratified analyses by sex (adjusted estimations)

eReferences.

This supplementary material has been provided by the authors to give readers additional information about their work.

© 2025 Martínez P et al. *JAMA Network Open*.

eTable 1. Distribution of cannabis use during adolescence in the response sample

During the past 12 months, how often did you use the following drug: cannabis (marijuana, hashish)?				
	**Age 12**	**Age 13**	**Age 15**	**Age 17**
I didn't use	1,342	1,187	1,096	748
Just once	5[a]	23	84	114
Less than once a month or occasionally	-	5	108	179
About once a month	-	5	47	62
During the weekend or 1-2 times a week	-	5	53	66
More than 3 times a week	-	7[b]	25	44
Every day	-	-	23	50

[a] To comply with ISQ participant identity-protection guidelines, "Just once" and "Less than once a month or occasionally" were collapsed into the "Just once" category.
[a] To comply with ISQ participant identity-protection guidelines, "More than 3 times a week" and "Every day" were collapsed into the "More than 3 times a week" category.

Data were compiled from the final master file of the Quebec Longitudinal Study of Child Development (1998–2023), ©Gouvernement du Québec, Institut de la Statistique du Québec.

© 2025 Martínez P et al. *JAMA Network Open*.

eTable 2. Diagnostic definitions and codes for mental, neurodevelopmental, and physical health conditions using administrative medical records

Condition	ICD-9 codes	ICD-10 codes	Definition
**Mental and behavioral disorders**			**If any common mental disorder, serious mental disorders, or susbtance-related disorders are recorded, as per the following definitions + "adult-onset" ADHD.**
**Common mental disorders**[1,2]			The diagnoses must have been recorded at least once during a hospital stay (MED-ECHO), at least once during an emergency room visit (BDCU [ICD-10 codes only]), or at least twice during separate fee-for-service visits (RAMQ). There were two classification periods: childhood (before 12 years of age [confounder]) and young adulthood (after 18 years of age [outcome]).
Depressive disorders[3,4]	300.4, 311, 311.9	F32.x, F33.x, F34.1, F34.8, F34.9, F38.x, F39, F41.2	
Anxiety disorders[4]	300.x [x != 4]	F40.x, F41.x [x != 2], F42.x, F45.x, F48.x	
Adjustment disorders[3,4]	309.x	F43.x	
**Serious mental disorders**[5,6]			The diagnoses must have been recorded at least once during a hospital stay (MED-ECHO), at least once during an emergency room visit (BDCU [ICD-10 codes only]), or at least once during a fee-for-service visits (RAMQ). There were two classification periods: childhood (before 12 years of age [confounder]) and young adulthood (after 18 years of age [outcome]).
Bipolar disorders[3]	296.x	F30.x, F31.x, F34.0	
Schizophrenia spectrum and other psychotic disorders[4,7]	295.x, 297.x, 298.1, 298.3, 298.4, 298.8, 298.9	F20.x, F21, F22.x, F23.x, F24, F25.x, F28, F29	

© 2025 Martínez P et al. *JAMA Network Open*.

**Substance-related disorders**			The diagnoses must have been recorded at least once during a hospital stay (MED-ECHO), at least once during an emergency room visit (BDCU [ICD-10 codes only]), or at least once during a fee-for-service visits (RAMQ). There were two classification periods: childhood (before 12 years of age [confounder]) and young adulthood (after 18 years of age [outcome]).
Alcohol-related disorders[8]	291.x, 303.x, 305.0, 357.5, 425.5, 535.3, 571.0-571.3, 980.0, 980.1, 980.8, 980.9	E24.4, F10.x, G31.2, G62.1, G72.1, I42.6, K29.2, K70.0-K70.4, K70.9, K85.2, K86.0, O35.4, T51.0, T51.1, T51.8, T51.9	
Cannabis-related disorders[4,8]	304.3, 305.2	F12.x, T40.7	
Other drug-related disorders[8]	292.x, 304.0-304.2, 304.4-304.9, 305.3-305.7, 965.0, 965.8, 967.0, 967.6, 967.8, 967.9, 969.4, 969.5, 969.7, 969.9, 970.8, 982.0, 982.8	F11.x, F12.x, F13.x, F14.x, F15.x, F16.x, F18.x, F19.x, T40.x [x != 7], T42.3, T42.4, T42.6, T42.7, T43.5, T43.6, T43.8, T43.9, T50.9, T52.8, T52.9	
**Suicide-related behaviors**[1,9–11]	E95.0-E95.9	X60-X84, Y87.0	The diagnoses must have been recorded at least once during a hospital stay (MED-ECHO), at least once during an emergency room visit (BDCU [**ICD-10 codes X & Y are NOT recorded**, **instead triage codes 0680 & 0681 for suicide-related behaviors in the 'Reason for the emergency visit' were used**]), or at least once during a fee-for-service visits (RAMQ [ICD-10 codes only, **ICD-9 'E' codes were not used in the RAMQ**]). **There was one classification period: young adulthood (after 18 years of age [outcome]).**

© 2025 Martínez P et al. *JAMA Network Open*.

**Neurodevelopmental disorders**[12–16]			The diagnoses must have been recorded at least once during a hospital stay (MED-ECHO), at least once during an emergency room visit (BDCU [ICD-10 codes only]), or at least twice during separate fee-for-service visits (RAMQ). **There was one classification period: childhood (before 12 years of age [confounder])**. The diagnoses must have been recorded at least once during a hospital stay (MED-ECHO), at least once during an emergency room visit (BDCU [ICD-10 codes only]), or at least twice during separate fee-for-service visits (RAMQ). There were two classification periods: childhood (before 12 years of age [confounder]) and young adulthood (after 18 years of age [outcome]). Within this classification, a further distinction is made to identify "adult-incident" (i.e., diagnosed) neurodevelopmental disorders.
Mental retardation	317.x, 318.x, 319.x	F70.x, F71.x, F72.x, F73.x, F78.x, F79.x	
Disorders of psychological development	299.x, 315.x	F80.x, F81.x, F82, F83, F84.x, F88, F89	
Attention deficit/hyperactivity disorder[17]	314.x	F90.x	
Stereotyped movement disorders	307.3	F98.4	
Tic disorders	307.2	F95.x	
**Disturbances of conduct and emotions occurring in childhood or adolescence**[12,15,16]	312.x, 313.x	F91.x, F92.x, F93.x, F94.1, F94.2, F94.8, F94.9	The diagnoses must have been recorded at least once during a hospital stay (MED-ECHO), at least once during an emergency room visit (BDCU [ICD-10 codes only]), or at least twice during separate fee-for-service visits (RAMQ). **There was one classification period: childhood (before 12 years of age [confounder])**.
**Physical conditions**			

© 2025 Martínez P et al. *JAMA Network Open*.

**Diseases of the respiratory system**[18,19]	460.x-519.x	J00-J99.x	The diagnoses must have been recorded at least once during a hospital stay (MED-ECHO), at least once during an emergency room visit (BDCU [ICD-10 codes only]), or at least once during a fee-for-service visits (RAMQ). There were two classification periods: childhood (before 12 years of age [confounder]) and young adulthood (after 18 years of age [outcome]).
Asthma	493.x	J45.x	
**Injuries and poisoning**[20–22]	800.x-995.x [!= 965.0, 965.8, 967.0, 967.6, 967.8, 967.9, 969.4, 969.5, 969.7, 969.9, 970.8, 980.0, 980.1, 980.8, 980.9, 982.0, 982.8, **E95.0-E95.9**]	S00.x-T79.x [!= T40.x, T42.3, T42.4, T42.6, T42.7, T43.5, T43.6, T43.8, T43.9, T50.9, T51.0, T51.1, T51.8, T51.9, T52.8, T52.9], T90.x-T98.x, [!= **X60-X84, Y87.0**]	The diagnoses must have been recorded at least once during a hospital stay (MED-ECHO), at least once during an emergency room visit (BDCU [**ICD-10 codes X & Y are NOT recorded**, **instead triage codes 0680 & 0681 for suicide-related behaviors in the 'Reason for the emergency visit' were used**]), or at least twice during separate fee-for-service visits (RAMQ [ICD-10 codes only, **ICD-9 'E' codes were not used in the RAMQ**]). **Importantly, during each one of these medical encounters, codes for suicide-related behaviors should not have been used.** There were two classification periods: childhood (before 12 years of age [confounder]) and young adulthood (after 18 years of age [outcome]).
**Other physical diseases**[19]			The diagnoses must have been recorded at least once during a hospital stay (MED-ECHO), at least once during an emergency room visit (BDCU [ICD-10 codes only]), or at least once during a fee-for-service visits (RAMQ). There were two classification periods: childhood (before 12 years of age [confounder]) and young adulthood (after 18 years of age [outcome]).
Infectious and parasitic diseases[18]	000.x-139.x	A00.x-A99, B00.x-B99, U04.x	

© 2025 Martínez P et al. *JAMA Network Open*.

Neoplasms[18]	140.x-239.x	C00.x-C97, D00.x-D48.x	
Diseases of the blood, blood-forming organs, and the immune mechanism	280.x-289.x, 279.x	D50.x-D89.x	
Endocrine, nutritional, and metabolic diseases[18]	240.x-278.x	E00.x-E90 [!= E24.4]	
Diseases of the nervous system and sense organs[18]	320.x-389.x [!= 357.5]	G00.x-G99.x [!= G62.1, G31.2, G72.1], H00.x-H95.x	
Diseases of the circulatory system[18]	390.x-459.x [!= 425.5]	I00-I99 [!= I42.6]	
Diseases of the digestive system[18]	520.x-579.x [!= 535.3, 571.0-571.3]	K00.x-K93.x [!= K70.0-K70.4, K70.9, K29.2, K85.2, K86.0]	
Diseases of the genitourinary system	580.x-629.x	N00.x-N99.x	
Diseases of the skin and subcutaneous tissue	680.x-709.x	L00.x-L99.x	
Diseases of the musculoskeletal system and connective tissue	710.x-739.x	M00.x-M99.x	

X represents a wildcard and can be replaced by any digit; != indicates codes or digits that should be excluded from a given definition.

Data were compiled from the final master file of the Quebec Longitudinal Study of Child Development (1998–2023), ©Gouvernement du Québec, Institut de la Statistique du Québec.

eAppenix 1. Selection of pre-exposure individual, family, and community-level confounders

Confounders were organized into nine pre-exposure domains based on temporal precedence and causal role: parental demographics, structural resources (household socioeconomic status and neighborhood characteristics), family psychosocial functioning (parental mental health and parenting practices), adverse childhood experiences, sex of the child, child proximal phenotypes (cognitive development, child's mental health, and child's social capabilities), peer influence, and prior medical care use. All domains precede the exposure (adolescent cannabis use) and can affect the outcome (medical care use), thereby acting as confounders. A full, visually dense, variable-level DAG and dagitty code are provided in: https://dagitty.net/dags.html?id=PcMhWBvJ.

1. **Parental demographics**, which define the child's family of origin and parental context (e.g., parental ethnicity, parental age at birth, household structure at the time of birth), shape early environments and opportunities. These are linked to household socioeconomic status (e.g., younger parental age disrupts parents' own schooling and career progression), parenting practices (e.g., single mothers' parenting practices may be compromised by several stressors), parental mental health (e.g., systemic racism elevates depressive symptoms among racialized individuals), neighborhood characteristics (e.g., parental ethnicity influences residential choices), child's social capabilities (e.g., parental ethnicity and associated socialization practices shape children's social skills), cognitive development (e.g., younger parents may have fewer resources to provide cognitively stimulating activities), previous history of medical care use (e.g., younger or minority parents may face barriers to accessing healthcare services early in a child's life), adolescent cannabis use (e.g., adolescents with parents from ethnic groups characterized by strong disapproval of cannabis use may be less likely to experiment), and medical care use (e.g., minority families may face barriers to care, including language challenges and cultural mistrust of healthcare systems).

2. **Structural resources**
   - 2.1. **Household socioeconomic status**, defined by parental education, income, and employment, determines access to resources and opportunities within the household. It is causally linked to parenting practices (e.g., higher socioeconomic status enables greater investment in enrichment activities and consistent caregiving, while lower status can limit supervision due to parental work demands), parental mental health (e.g., financial instability increases the likelihood of stress, depression, and anxiety), neighborhood characteristics (e.g., socioeconomic status determines the affordability of housing in safer or more resource-rich neighborhoods), previous history of medical care use (e.g., higher socioeconomic status increases access to preventive healthcare, while lower socioeconomic status may delay necessary care), adolescent cannabis use (e.g., lower socioeconomic status may increase exposure to environments with permissive norms around substance use or reduce access to preventive interventions), and medical care use (e.g., families with higher socioeconomic status are more likely to afford healthcare services, while lower status may delay or reduce utilization due to financial barriers).
   - 2.2. **Neighborhood characteristics** refer to the socioeconomic and physical environment surrounding the child's home, including factors such as neighborhood safety, access to resources (e.g., schools, parks, healthcare), and the socioeconomic status of the area. These are causally linked to parenting practices (e.g., unsafe neighborhoods may lead to stricter supervision or limited outdoor activities), child's mental health (e.g., exposure to violence or resource deprivation increases stress and emotional difficulties), peer influence (e.g., neighborhood composition shapes the availability of supportive or risky peer groups), previous history of medical care use (e.g., neighborhoods with better healthcare access lead to higher early utilization), adolescent cannabis use (e.g., neighborhoods with high substance use prevalence increase the likelihood of exposure and initiation), and medical care use (e.g., proximity to healthcare facilities facilitates or limits access to services).

3. **Family psychosocial functioning**

© 2025 Martínez P et al. *JAMA Network Open*.

3.1. **Parental mental health** refers to the psychological well-being of the parents, including conditions such as stress, anxiety, depression, and substance use or abuse. This is causally linked to parenting practices (e.g., parental depression or substance abuse reduces emotional availability and consistency in discipline), adverse childhood experiences (e.g., untreated mental health conditions or substance abuse increase exposure to neglect, household conflict, or unsafe environments), child's mental health (e.g., parental anxiety or substance use increases the likelihood of emotional regulation difficulties in children), previous history of medical care use (e.g., untreated parental mental health issues may result in neglected child healthcare or more frequent visits related to family stressors), adolescent cannabis use (e.g., parental stress or substance abuse may decrease supervision or normalize substance use, increasing the risk of initiation), and medical care use (e.g., parents with mental health or substance use challenges are less likely to ensure timely healthcare access for their children due to neglect or diminished organizational capacity).

3.2. **Parenting practices**, encompassing supervision, discipline, and caregiving behaviors, shape the child's developmental environment. These are causally linked to child's social capabilities (e.g., encouraging prosocial behaviors fosters stronger peer relationships), peer influence (e.g., monitoring limits exposure to deviant peers), child's mental health (e.g., consistent caregiving reduces the risk of emotional or behavioral issues), adverse childhood experiences (e.g., inconsistent discipline increases exposure to neglect or conflict), cognitive development (e.g., engaging parenting supports problem-solving skills), adolescent cannabis use (e.g., strong parental oversight reduces substance initiation), and medical care use (e.g., attentive caregiving ensures timely healthcare access).

4. **Adverse childhood experiences** refer to potentially traumatic events or circumstances experienced during childhood, such as abuse, neglect, or household dysfunction (e.g., parental substance use or domestic violence). These are causally linked to parenting practices (e.g., parental conflict or household dysfunction exacerbate inconsistent caregiving behaviors), child's mental health (e.g., adverse childhood experiences increase the risk of emotional and behavioral problems), peer influence (e.g., children with adverse childhood experiences may gravitate toward deviant peer groups due to emotional dysregulation), cognitive development (e.g., adverse childhood experiences impair learning and problem-solving abilities due to chronic stress), previous history of medical care use (e.g., children experiencing abuse or neglect often require medical attention for physical injuries or psychological support), adolescent cannabis use (e.g., adverse childhood experiences increase susceptibility to substance use as a coping mechanism), and medical care use (e.g., children with adverse childhood experiences often require more healthcare services for physical and mental health issues).

5. **Sex of the child**, determined at birth, refers to the child's biological designation as male or female. This is causally linked to parenting practices (e.g., parents often impose stricter monitoring on girls and encourage more independence in boys), child's mental health (e.g., boys are more likely to develop externalizing behaviors such as aggression, while girls exhibit internalizing behaviors such as anxiety), peer influence (e.g., boys typically form activity-based peer groups, while girls prioritize relational groups that influence social behaviors), adolescent cannabis use (e.g., boys have a higher likelihood of engaging in risk-taking behaviors, including substance use), and medical care use (e.g., boys more frequently require care for injuries, while girls are more likely to access mental health and reproductive health services).

4. **Child proximal phenotypes**

4.1. **Cognitive development** refers to the child's intellectual growth and academic readiness, including skills such as language acquisition, problem-solving, and attention regulation. This is causally linked to child's social capabilities (e.g., cognitive abilities enhance the child's adaptability and conflict resolution in social interactions), child's mental health (e.g., strong cognitive skills improve emotional regulation and reduce behavioral problems), peer influence (e.g., academically skilled children are more likely to associate with prosocial

© 2025 Martínez P et al. *JAMA Network Open*.

peers), adolescent cannabis use (e.g., lower cognitive functioning may increase vulnerability to risk-taking behaviors, including substance use), and medical care use (e.g., cognitive delays may necessitate specialized interventions or therapies).

4.2. Child's mental health refers to the emotional, psychological, and behavioral well-being of the child, including internalizing behaviors (e.g., anxiety and depression) and externalizing behaviors (e.g., aggression or hyperactivity). This is causally linked to peer influence (e.g., children with behavioral challenges may gravitate toward deviant peer groups), adverse childhood experiences (e.g., mental health issues may intensify the impact of neglect or household conflict), cognitive development (e.g., behavioral and emotional problems can impair focus and learning), adolescent cannabis use (e.g., emotional or behavioral difficulties increase the likelihood of using cannabis as a coping mechanism), and medical care use (e.g., children with mental health issues require more frequent healthcare services, including therapy or medication).

4.3. Child's social capabilities capture the child's intrinsic abilities to engage with peers, including social skills, adaptability, and conflict resolution. It reflects the child's readiness to form and maintain relationships, as well as their ability to navigate social interactions successfully. This is causally linked to peer influence (e.g., strong social skills increase exposure to prosocial peer groups), child's mental health (e.g., poor social skills increase the risk of emotional and behavioral problems), adolescent cannabis use (e.g., children with social difficulties may turn to substance use as a coping mechanism), and medical care use (e.g., challenges in social adjustment may require therapeutic interventions or stress management support).

5. **Peer influence** reflects the child's external social context, including experiences such as peer victimization, rejection, or inclusion in supportive or risky peer groups. It accounts for the quality of the child's peer relationships and the broader social dynamics they are exposed to. This is causally linked to child's social capabilities (e.g., supportive peers enhance adaptability and conflict resolution skills), child's mental health (e.g., peer victimization increases stress, anxiety, and emotional distress), adolescent cannabis use (e.g., association with peers engaging in risky behaviors increases the likelihood of substance use), and medical care use (e.g., experiences of rejection or exclusion may result in a greater need for psychological or medical interventions).

6. **Prior medical care use** refers to the frequency and type of healthcare services utilized by the child or their family prior to the exposure period, including visits for routine checkups, illnesses, or injuries. This is causally linked to child's mental health (e.g., frequent medical interventions may increase awareness of mental health needs), cognitive development (e.g., early interventions for health or developmental issues can enhance or impair learning outcomes), adolescent cannabis use (e.g., early exposure to healthcare providers discussing risks may influence future behavior), and medical care use during adolescence and adulthood (e.g., established care-seeking behaviors in childhood often persist into later stages of life).

© 2025 Martínez P et al. *JAMA Network Open*.

eFigure 1. Directed acyclic graph – Developmental pathways to adolescent cannabis use and utilization of medical care

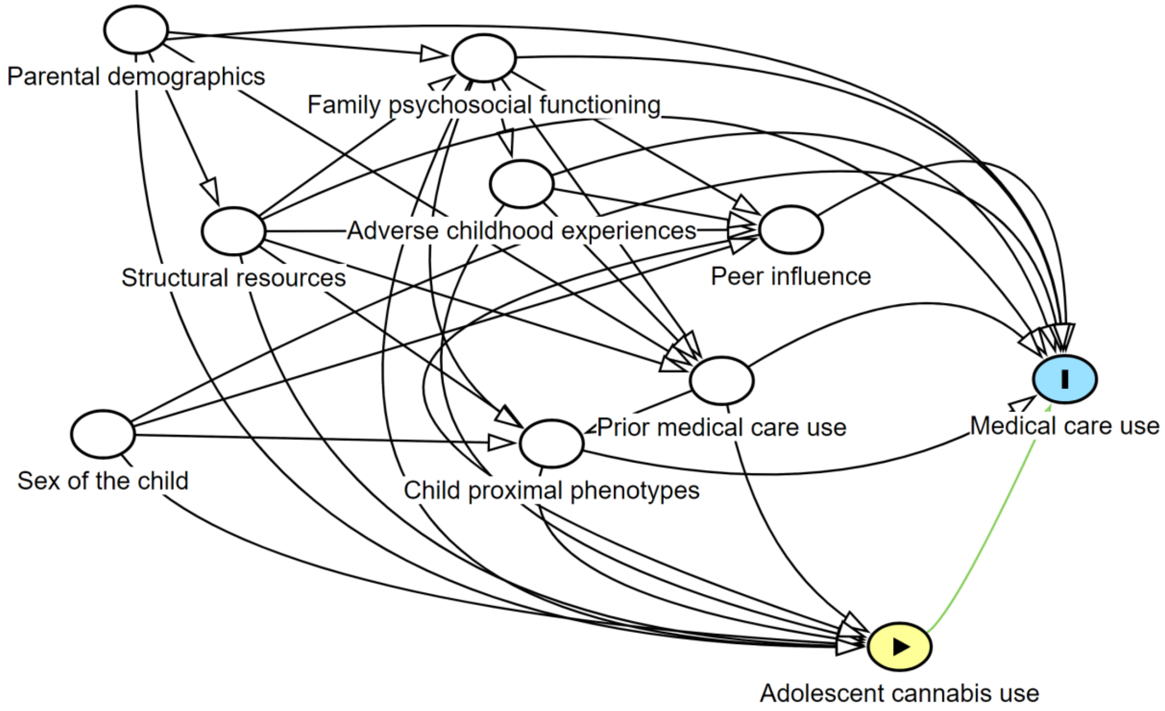

Permanent URL: https://dagitty.net/dags.html?id=quM8a2aQ.

© 2025 Martínez P et al. *JAMA Network Open*.

eTable 3. Final parameter estimates from the group-based trajectory model

Trajectory group	Polynomial order	Intercept	Linear term	Quadratic term
Non-users	0	-6.54	-	-
Late-onset users	1	-21.77	1.32	-
Early-onset & frequent users	2	-64.81	8.20	-0.25

P-values for all parameters were < 0.001.

Data were compiled from the final master file of the Quebec Longitudinal Study of Child Development (1998–2023), ©Gouvernement du Québec, Institut de la Statistique du Québec.

eTable 4. Patterns of cannabis use during adolescence and medical care utilization for mental or physical health conditions between ages 18 to 23 in the response sample

	Unadjusted Odds Ratio (95% Confidence Interval)	Adjusted Odds Ratio (95% Confidence Interval)
**Mental health**		
Any mental disorder		
Non-users	1 (reference)	1 (reference)
Late-onset users	1.12 (0.82 to 1.52)	1.18 (0.78 to 1.77)
Early-onset and frequent users	**1.65 (1.24 to 2.19)**	**1.73 (1.15 to 2.59)**
Common mental disorders		
Non-users	1 (reference)	1 (reference)
Late-onset users	1.05 (0.75 to 1.46)	1.14 (0.73 to 1.78)
Early-onset and frequent users	**1.70 (1.26 to 2.29)**	**1.70 (1.10 to 2.61)**
Any substance-related disorder		
Non-users	1 (reference)	1 (reference)
Late-onset users	1.05 (0.58 to 1.89)	1.01 (0.48 to 2.12)
Early-onset and frequent users	**1.95 (1.20 to 3.17)**	**2.86 (1.51 to 5.43)**
Suicide-related behaviors		
Non-users	1 (reference)	1 (reference)
Late-onset users	0.96 (0.48 to 1.91)	0.75 (0.27 to 2.09)
Early-onset and frequent users	**1.82 (1.04 to 3.20)**	1.25 (0.54 to 2.90)
**Physical health conditions**		
Any physical condition		
Non-users	1 (reference)	1 (reference)
Late-onset users	**1.64 (1.21 to 2.22)**	**1.61 (1.06 to 2.44)**
Early-onset and frequent users	**2.07 (1.50 to 2.85)**	**1.79 (1.13 to 2.85)**
Respiratory diseases		
Non-users	1 (reference)	1 (reference)
Late-onset users	1.01 (0.73 to 1.38)	1.08 (0.70 to 1.66)
Early-onset and frequent users	**1.43 (1.07 to 1.92)**	1.32 (0.87 to 2.00)
Injuries and poisoning		
Non-users	1 (reference)	1 (reference)
Late-onset users	1.28 (0.98 to 1.68)	1.09 (0.76 to 1.57)
Early-onset and frequent users	**1.63 (1.25 to 2.12)**	1.31 (0.90 to 1.90)
Other physical diseases		
Non-users	1 (reference)	1 (reference)
Late-onset users	**1.33 (1.02 to 1.73)**	1.28 (0.89 to 1.83)
Early-onset and frequent users	**1.65 (1.26 to 2.16)**	**1.49 (1.01 to 2.19)**

Statistically significant estimates are shown in bold. Inverse-probability weightings were applied to account for sampling differences between the study sample and the complete cohort. Adjusted estimations used overlap weighting to address confounder imbalance.
Further details on outcome definitions can be found in **eTable 2**.
Data were compiled from the final master file of the Quebec Longitudinal Study of Child Development (1998–2023), ©Gouvernement du Québec, Institut de la Statistique du Québec.

eFigure 2. Confounder balance in the response sample

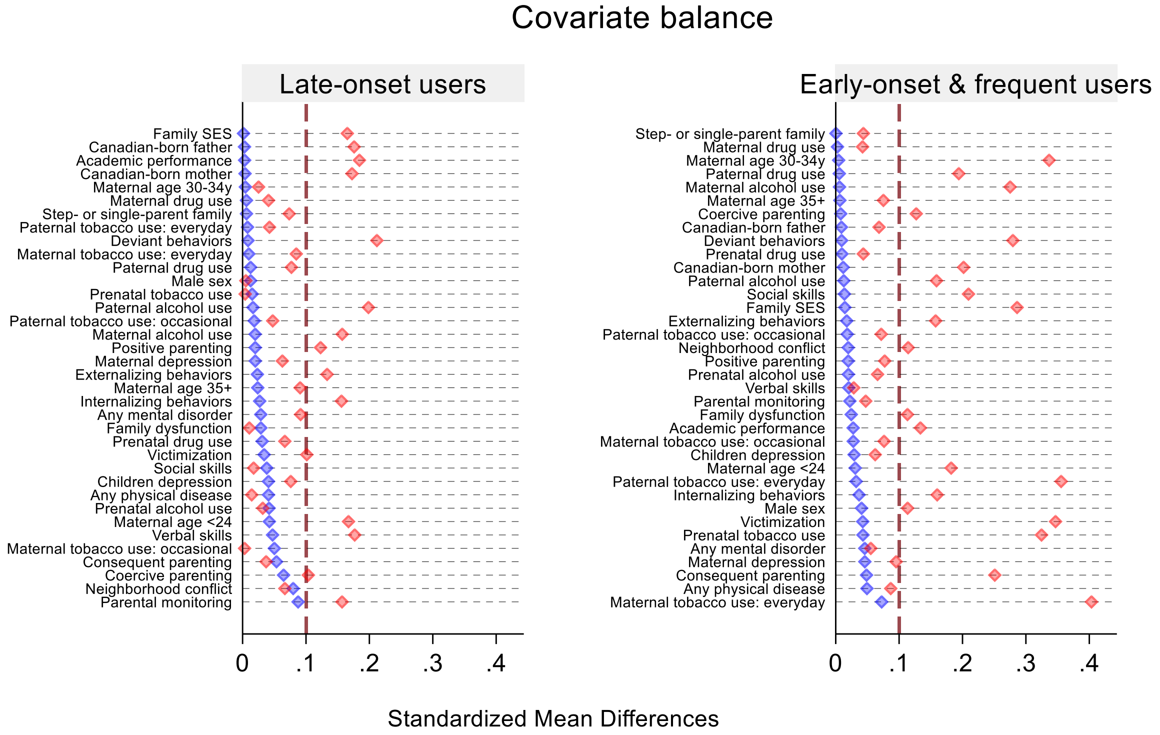

The plot displays the absolute (pairwise) standardized mean differences for comparisons between the exposed groups (early-onset and frequent users, and late-onset users) and the non-exposed group (non-users) in the response sample. The dashed line denotes the commonly accepted threshold of 0.1 for standardized mean differences, indicating adequate confounder balance. Red diamonds show the unweighted estimates, whereas blue diamonds represent the overlap-weighted estimates (i.e., after applying overlap weights to balance confounders).
Data were compiled from the final master file of the Quebec Longitudinal Study of Child Development (1998–2023), ©Gouvernement du Québec, Institut de la Statistique du Québec.

eAppendix 2. interaction analyses for sex, tobacco use, and alcohol use

We conducted additional interaction analyses to assess whether the associations between adolescent cannabis use and medical care utilization for any mental disorder or any physical condition differed by sex, tobacco use, or alcohol use. For any mental disorder by sex, the interaction odds ratios (ORs) for late-onset and early-onset and frequent cannabis users were 0.90 (95% CI, 0.45–1.79) and 0.93 (95% CI, 0.48–1.78), respectively, whereas for any physical condition by sex, they were 1.33 (95% CI, 0.68–2.62) and 0.70 (95% CI, 0.34–1.46). When examining tobacco use, the ORs for any mental disorder were 2.33 (95% CI, 0.76–7.16) for late-onset users and 0.66 (95% CI, 0.26–1.68) for early-onset and frequent users, and for any physical condition, 1.07 (95% CI, 0.30–3.82) and 0.49 (95% CI, 0.18–1.35), respectively. Regarding alcohol use, the ORs for any mental disorder were 1.04 (95% CI, 0.62–1.76) for late-onset users and 0.98 (95% CI, 0.65–1.50) for early-onset/and frequent users, and for any physical condition, 0.87 (95% CI, 0.53–1.43) and 0.77 (95% CI, 0.48–1.23). Because all 95% CIs included 1, no statistically significant interaction was detected. These findings suggest that the increased medical care utilization observed among early-onset and frequent cannabis users is consistent across sex and does not vary significantly with adolescent tobacco or alcohol use.

Complementarily, sex-stratified results suggest that both males and females who used cannabis earlier and frequently during adolescence are vulnerable to increased medical care utilization for any physical health condition, whereas the elevated risk of medical care utilization for mental disorders appears more pronounced among females engaging in early-onset and frequent use (eTable 5).

© 2025 Martínez P et al. *JAMA Network Open*.

eTable 5. Stratified analyses by sex (adjusted estimations)

	**Males Odds Ratio (95% Confidence Interval)**	**Females Odds Ratio (95% Confidence Interval)**
Any mental disorder		
Non-users	1 (reference)	1 (reference)
Late-onset users	1.06 (0.63 to 1.78)	1.18 (0.75 to 1.85)
Early-onset and frequent users	1.46 (0.89 to 2.41)	**1.58 (1.04 to 2.39)**
Any physical condition		
Non-users	1 (reference)	1 (reference)
Late-onset users	**1.86 (1.17 to 2.98)**	1.40 (0.86 to 2.27)
Early-onset and frequent users	**1.66 (1.03 to 2.67)**	**2.35 (1.35 to 4.10)**

Estimates are adjusted odds ratios and 95% confidence intervals.
Statistically significant estimates are shown in bold. Inverse-probability weightings were applied to account for sampling differences between the study sample and the complete cohort. Adjusted estimations using overlap weighting to address confounder imbalance are shown in blue.
Data were compiled from the final master file of the Quebec Longitudinal Study of Child Development (1998–2023), ©Gouvernement du Québec, Institut de la Statistique du Québec.

© 2025 Martínez P et al. *JAMA Network Open*.

eReferences

1. Gobbi G, Atkin T, Zytynski T, Wang S, Askari S, Boruff J, et al. Association of Cannabis Use in Adolescence and Risk of Depression, Anxiety, and Suicidality in Young Adulthood: A Systematic Review and Meta-analysis. JAMA Psychiatry. 2019 Apr 1;76(4):426–34.

2. Lowe DJE, Sorkhou M, George TP. Cannabis use in adolescents and anxiety symptoms and disorders: a systematic review and meta-analysis. Am J Drug Alcohol Abuse. 2024 Mar 3;50(2):150–61.

3. Leclerc J, Lesage A, Rochette L, Huỳnh C, Pelletier É, Sampalis J. Prevalence of depressive, bipolar and adjustment disorders, in Quebec, Canada. J Affect Disord. 2020 Feb 15;263:54–9.

4. Fleury MJ, Cao Z, Armoon B, Grenier G, Lesage A. Profiles of patients using emergency departments or hospitalized for suicidal behaviors. Suicide Life Threat Behav. 2022;52(5):943–62.

5. Matheson SL, Laurie M, Laurens KR. Substance use and psychotic-like experiences in young people: a systematic review and meta-analysis. Psychol Med. 2023 Jan;53(2):305–19.

6. Jefsen OH, Erlangsen A, Nordentoft M, Hjorthøj C. Cannabis Use Disorder and Subsequent Risk of Psychotic and Nonpsychotic Unipolar Depression and Bipolar Disorder. JAMA Psychiatry. 2023 Aug 1;80(8):803–10.

7. Vanasse A, Courteau J, Fleury MJ, Grégoire JP, Lesage A, Moisan J. Treatment prevalence and incidence of schizophrenia in Quebec using a population health services perspective: different algorithms, different estimates. Soc Psychiatry Psychiatr Epidemiol. 2012 Apr 1;47(4):533–43.

8. Huỳnh C, Kisely S, Rochette L, Pelletier É, Jutras-Aswad D, Larocque A, et al. Using administrative health data to estimate prevalence and mortality rates of alcohol and other substance-related disorders for surveillance purposes. Drug Alcohol Rev. 2021 May;40(4):662–72.

9. Rahme E, Low NCP, Lamarre S, Daneau D, Habel Y, Turecki G, et al. Correlates of Attempted Suicide from the Emergency Room of 2 General Hospitals in Montreal, Canada. Can J Psychiatry Rev Can Psychiatr. 2016 July;61(7):382–93.

10. Levesque P, Perron PA. Les comportements suicidaires au Québec : Portrait 2024. Québe. Québec: , Bureau d'information et d'études en santé des populations, Institut national de santé publique du Québec; 2024 p. 71. Report No.: 3451.

11. Silins E, Horwood LJ, Patton GC, Fergusson DM, Olsson CA, Hutchinson DM, et al. Young adult sequelae of adolescent cannabis use: an integrative analysis. Lancet Psychiatry. 2014 Sept 1;1(4):286–93.

12. Groenman AP, Janssen TWP, Oosterlaan J. Childhood Psychiatric Disorders as Risk Factor for Subsequent Substance Abuse: A Meta-Analysis. J Am Acad Child Adolesc Psychiatry. 2017 July 1;56(7):556–69.

13. Cawkwell PB, Hong DS, Leikauf JE. Neurodevelopmental Effects of Cannabis Use in Adolescents and Emerging Adults with ADHD: A Systematic Review. Harv Rev Psychiatry. 2021;29(4):251–61.

© 2025 Martínez P et al. *JAMA Network Open*.

14. Artigas MS, Sánchez-Mora C, Rovira P, Richarte V, García-Martínez I, Pagerols M, et al. Attention-Deficit/Hyperactivity Disorder and lifetime cannabis use: genetic overlap and causality. Mol Psychiatry. 2020 Oct;25(10):2493–503.

15. Fergusson DM, Horwood LJ, Ridder EM. Conduct and attentional problems in childhood and adolescence and later substance use, abuse and dependence: results of a 25-year longitudinal study. Drug Alcohol Depend. 2007 Apr;88 Suppl 1:S14-26.

16. Heron J, Barker ED, Joinson C, Lewis G, Hickman M, Munafò M, et al. Childhood conduct disorder trajectories, prior risk factors and cannabis use at age 16: birth cohort study. Addict Abingdon Engl. 2013 Dec;108(12):2129–38.

17. Surveillance du trouble du déficit de l'attention avec ou sans hyperactivité (TDAH) au Québec. Québec: INSPQ, Institut national de santé publique du Québec, BIESP, Bureau d'information et d'études en santé des populations; 2019.

18. Huynh C, L'Esperánce N, Rochette L, Fleury MJ, Jutras-Aswad D, Kisely S, et al. Substance-related disorders: mortality surveillance. Québec: Institut national de santé publique du Québec; 2023 p. 34. Report No.: 3311.

19. Tuvel AL, Winiger EA, Ross JM. A Review of the Effects of Adolescent Cannabis Use on Physical Health. Child Adolesc Psychiatr Clin N Am. 2023 Jan;32(1):85–105.

20. R B, J S, D MF, A L, R L. A method for deriving leading causes of death. Bull World Health Organ [Internet]. 2006 Apr [cited 2025 July 26];84(4). Available from: https://pubmed.ncbi.nlm.nih.gov/16628303/

21. Koivisto MK, Puljula J, Levola JM, Mustonen A, Miettunen J, Alakokkare AE, et al. Adolescent alcohol and cannabis use as risk factors for head trauma in the Northern Finland Birth Cohort study 1986. Eur J Public Health. 2023 Dec 1;33(6):1115–21.

22. The Health Effects of Cannabis and Cannabinoids: The Current State of Evidence and Recommendations for Research [Internet]. Washington, D.C.: National Academies Press; 2017 [cited 2024 Feb 15]. Available from: https://www.nap.edu/catalog/24625

23. Chaplin TM, Aldao A. Gender differences in emotion expression in children: A meta-analytic review. Psychol Bull. 2013;139(4):735–65.

© 2025 Martínez P et al. *JAMA Network Open*.
