# Supplementary material for: Health Service Use Among Young Adults With a History of Adolescent Cannabis Use
Source: JAMA Netw Open. 2025 Oct 28;8(10):e2539977. doi: 10.1001/jamanetworkopen.2025.39977 (PMC12569714; doi:10.1001/jamanetworkopen.2025.39977)
Supplement: Supplement 2. — Data Sharing Statement [file jamanetwopen-e2539977-s002.pdf]

Data Sharing Statement

Martínez. Health Service Use Among Young Adults With a History of Adolescent Cannabis Use. *JAMA Netw Open*. Published October 28, 2025.
doi:10.1001/jamanetworkopen.2025.39977

Data
Data available: No

Additional Information
Explanation for why data not available: Available to authorized researchers
